# The Book World of Medicine and Science

**Published:** 1903-01-31

**Authors:** 


### THE BOOK WORLD OF MEDICINE AND SCIENCE.

A TEXT-BOOK OF THE SCIENCE AND ART OF OBSTETRICS By HENRY J. GARRIGUES, A.M., M.D., Consulting Obstetric Surgeon to the New York Maternity Hospital. With 504 Illustrations. (Philadelphia and London: J. B. Lippincott Co. 1902. Price 25s. net.)

DR. GARRIGUES has produced an admirable text-book in which the student will find practically all that he requires in regard to the science and art of obstetrics, although so largely does the practice of midwifery in abnormal cases verge upon gynæcology that he will probably have to refer occasionally to works on the latter specialty for the full elucidation of problems which may arise from time to time. The book before us is divided in a useful and practical manner into two parts, the one dealing with the normal and the other with the abnormal. Naturally the normal comes first. The interesting processes involved in the development of the ovum and the accompanying alterations in the uterine and other maternal organs are well and carefully described, many details being entered into perhaps more fully than is usual in some text-books. Month by month the history of events is traced, and many hints are given as to management of the patient during this time, until the period of maturity arrives and we are brought by easy stages to the subject of normal labour. Here, beginning with a complete description of the anatomy of the parts and of the forces engaged, a fairly good account is given of the events which characterise the process of child-birth and rules are laid down for the safe conduct of each step in the conduct of the case. Curiously little stress is laid upon the various "presentations," and we rather miss the familiar diagrams describing the mechanism of labour and the varying positions of the fœtal head in its different stages. Truly we have Tarnier's many-headed illustration of "rotation" and "extension," but we doubt whether such fully-shaded "picture" illustrations are as instructive as the old-fashioned diagrams showing the varying position of the sutures and fontanelles. Then come chapters on midwives and on lying-in institutions, and on the normal puerpery in regard to both mother and child, which concludes the portion dealing with the normal. The remaining two-thirds of the book are devoted to abnormal pregnancy and labour, to the treatment of the various complications and emergencies of which they form a useful guide. The chapters on obstetric operations also contain good descriptions of the procedures to be adopted. Special attention is given to symphysiotomy, which is described in considerable detail. In summing up the matter Dr. Garrigues says that "symphysiotomy ought to replace craniotomy on the living fœtus whenever it is possible to perform the former. Under such circumstances it would be next to murder to kill the fœtus, and even craniotomy, which deliberately destroys one of the two lives at stake in a delivery, is not without danger to the other," and he goes on to say that in addition to other indications, "even difficult forceps and version operations ought to be replaced by symphysiotomy." We cannot but think that Dr. Garrigues makes somewhat too light of certain possible after effects when he says that "if there are no complications, the patient may safely leave the bed at the end of three weeks, and be dismissed a week later. Perhaps the period of rest may even be shortened to two weeks." He admits, however, that "nobody should undertake this operation who is not an operative gynæcologist or a general surgeon with obstetric experience;" also that "the operator must have proper assistance," three assistants being indispensable and an extra one, capable of acting as either surgeon or accoucheur, being desirable, since "in some cases serious hæmorrhage has to be checked, in others serious injuries of delicate and important organs demand immediate repair;" that it is next to impossible to perform an aseptic operation in most private dwellings; and that "the after treatment is often quite complicated." Hence it is evident that symphysiotomy, whatever may be its advantages when performed at the proper moment amid complete hospital surroundings, is a thing for the private practitioner to adventure upon with a certain diffidence. We have read this book with much interest, and we feel no doubt that anyone who will carefully study its pages will obtain a good working knowledge of the subject. There are in it, however, certain omissions, from an English examiner's point of view, which make us doubt whether it will largely displace certain well-known English manuals among students in this country.

ANNUAL REPORT OF THE METROPOLITAN ASYLUMS BOARD, 1901. In two volumes. (London: McCorquodale and Company. 1902. Price 5s.)

THE first of these two volumes contains the report of the Board and of its committees, while the second contains that of the Statistical Committee, together with a series of articles by the medical officers attached to the various hospitals. Among the many interesting matters dealt with small-pox and vaccination take a leading place, and in regard to these matters the Board takes some pains to defend the statistics which have been issued by it from time to time against the attacks which have been made upon their veracity. Besides the portions of the report dealing with hospitals and infectious diseases, there is a great deal of interesting matter concerning the other multifarious branches of activity with which the Asylums Board is occupied, such as imbecile asylums, training ships, ringworm schools, remand homes, defective children, and other topics. Much credit is due to the members of the Board whose work has evidently involved much labour and self-sacrifice; nor must we forget the permanent officials, for there is no doubt that to their intelligence and devotion must be attributed much of the success of the work which is described in this report.

MEDICAL, SURGICAL, AND PATHOLOGICAL REPORTS OF THE ROYAL SOUTHERN HOSPITAL, LIVERPOOL. Edited by G. P. NEWBOLT, F.R.C.S., C. J. MACALISTER, M.D., and LYN DIMOND, M.B., Ch.B. (Liverpool: Rockliff Bros. 1902.)

THIS, the first annual clinical and pathological report of the Royal Southern Hospital, Liverpool, shows that the staff of this institution is doing good and careful work. First come a series of somewhat elementary statistics in regard to the cases treated, and then follow various papers by the members of the hospital staff. The papers are far better than the statistics.

